# Qualitative exploration of perceived barriers of exclusive breastfeeding among pregnant teenagers in the Greater Accra Region of Ghana

**DOI:** 10.1186/s12889-022-14277-4

**Published:** 2022-10-10

**Authors:** Angela Kwartemaa Acheampong, Makombo Ganga-Limando, Lydia Aziato

**Affiliations:** 1 School of Nursing and Midwifery, Wisconsin International University College, Accra, Ghana; 2 Department of Health Studies, University of South Africa, Pretoria, South Africa; 3 University of Health and Allied Sciences, Ho, Ghana

**Keywords:** Ghana, Exclusive breastfeeding, Breastfeeding, Teenage mothers and teenage pregnancy

## Abstract

**Background:**

The World Health Organization endorses exclusive breastfeeding for the first six months of every child’s life since exclusive breastfeeding has the potential of saving thousands of infants’ lives. The global exclusive breastfeeding rate among mothers is sub-optimal. This predisposes infants born to teenage mothers to all types of ailments. Therefore, this study explored the factors that inhibit the practice of exclusive breastfeeding as perceived by pregnant teenagers in the Greater Accra Region of Ghana which is an urban area.

**Methods:**

The study used techniques in qualitative descriptive exploration to collect data from 30 pregnant teenagers through focus group discussions. Six focus group discussions were conducted and each group was made up of five participants. Informed consent was obtained from participants who were 18 years and above as well as parents of participants below 18 years while informed assent was obtained from participants below 18 years after purposive sampling. Interviews were audiotaped, transcribed and data were analysed through content analysis.

**Results:**

Two major themes and eight sub themes emerged from the data after analysis. Personal related barriers (negative emotional feelings, irrational thinking, perceived health risks to the baby and perceived self-inefficacy) and social related barriers (provider-client interaction, disapproval of exclusive breastfeeding by close relatives, unfriendly workplace policies and social myths) were the perceived factors that discouraged exclusive breastfeeding among teenage mothers.

**Conclusion:**

Health professionals should be trained to provide culturally sensitive care to teenage mothers in order to promote exclusive breastfeeding. The media, religious leaders and politicians should help debunk misconceptions about breastfeeding expressed by participants in the study.

## Background

There is a global recognition of the importance of breastfeeding by public policy-makers. The World Health Assembly Resolution 65.6 of 2012 requested countries to escalate exclusive breastfeeding rates in the first six months to at least 50.0% by the year 2025 [1]. According to the World Health Organization, practicing optimal breastfeeding between the ages of 0–23 months could reduce about eight hundred thousand (800,000) deaths among children under five years of age [1]. Exclusive breastfeeding is a cost effective strategy which ensures the survival of infants irrespective of gender, geographical location and race of the infant [2]. Between 2010 and 2018, the global weighted average exclusive breastfeeding rate for children at 4 to 5 months was 32% [3]. Within that same period, Africa recorded a weighted average of 37.3% [3]. The West and Central African countries recorded the lowest rate of 28% compared with 54% for the East and Southern African countries in 2014 [4]. In 2008, the exclusive breastfeeding rate in Ghana was 62% but that rate dropped to 53% in 2014 [5].

Globally, the World Health Organization (WHO) identified the lack of knowledge on the benefits of exclusive breastfeeding, aggressive promotion of breast milk substitute, lack of adequate skilled support, unsupportive breastfeeding health care practices and policies, inadequate maternity and paternity leave legislation as the main barriers of exclusive breastfeeding during the first six months [6]. Similarly, studies across the world [7–22] identified tight work schedules, lack of or minimal support from health workers, maternal stress, mode of deliveries, negative attitude towards breastfeeding, lack of breastfeeding role models, inadequate spousal/family support, employment restrictions and inadequate information from health professionals as barriers of exclusive breastfeeding up to six months post-delivery. In largely patriarchal societies like Indonesia, Nigeria and Ghana, paternal grandmothers have been cited as having a lot of influence on a mother’s ability to continue breastfeeding [23–25]. Most of these grandmothers have been reported to discourage breastfeeding in their societies and due to their powerful influences, those mothers could not breastfeed continually as recommended [26–28].

Teenage pregnancy is one of the major challenges facing the healthcare professionals in Ghana. Official report in 2015 [29] indicated that 14% of Ghanaian girls aged 15 to 19 years had started childbearing at the time of data collection. Motherhood can be stressful, challenging and rough, especially when the mother is a teenager as most of them give birth out of marriages to fathers who are equally unprepared and immature [30, 31].

Studies on breastfeeding intentions and early breastfeeding practices among adolescents showed that they are less likely to practice exclusive breastfeeding successfully than their older counterparts [32, 33]. It is therefore important to look at exclusive breastfeeding practices among teenage mothers in Ghana. It is against the above context that this study was conducted with the purpose of exploring perceived barriers to the practice of exclusive breastfeeding among teenage mothers in Ghana.

## Methodology

### Design and setting

The research design allowed the researchers to explore and describe the phenomenon of interest from the perspectives of the participants [34]. The study was conducted in the Greater Accra Region of Ghana. Greater Accra is the second most populated region after the Ashanti Region, with a population of 4,010,054, accounting for 15.4 per cent of Ghana’s total population. Teenagers make up 21% of the total population [35]. Participants consisted of pregnant teenagers attending antenatal care and delivery services at the public hospitals and polyclinics within the Accra Metropolis of Ghana.

## Sampling and data Collection procedures

Non-probability purposive sampling was used to select 30 pregnant teenagers between the ages of 13 and 19 years to participate in the study based on specified inclusion criteria. Pregnant teenagers who had attended at least three antenatal care visits prior to data collection period were included in the study. This criteria ensured that, the participants had received education on exclusive breastfeeding since education on exclusive breastfeeding starts immediately when a woman starts antenatal care. In addition, participants were asked if they had received education on exclusive breastfeeding before they were recruited into the study. Data were collected through focus group discussions. Six focus groups were involved in the study because data saturated on the sixth group. Each group had 5 participants. All the participants could speak English or Twi (main Ghanaian local language). A semi structured interview guide with two main sections (A & B) was used to assist in data collection. Section A was made up of questions about the socio-demographic characteristics of the participants in the study. Section B was made up of questions based on participants’ perspectives on barriers of exclusive breastfeeding for at least six months post-delivery. The researchers prevented biases by avoiding leading questions that would lead to particular answers in the conduct of the focus group discussions. The discussions were conducted at secluded areas at the hospitals with privacy. Discussions started with informal interactions that created relaxed atmospheres before core questions were asked. In addition, there were recapitulation of group rules before discussions started. Techniques that ensured within group interactions were used to elicit succinct responses from participants. Therefore, participants freely expressed themselves. Two of the focus group discussions were conducted in Twi whereas the other four were conducted in English. Each focus group discussion lasted between an hour and half and two hours. Notes were taken during each discussion. Discussions were audiotaped and transcribed verbatim from audio to text. Data collected in the local language were translated into English before transcription. Data collection was stopped after six focus group discussions since data saturation had been achieved at that point. The venues and times of the discussions were at the convenience of the participants.

### Data Analysis

The researchers used content analyses to process the generated data [34]. Data were analysed using inductive reasoning. Initially, transcripts were read several times by all three authors independently to understand the general perspectives of participants before names and phrases were attached to sentences (coding). The codes attached to sentences and phrases were the ones that captured the meanings of the data. After that, the authors met on different occasions and compared their independent codes to agree on a final codebook. Similar codes were then grouped together to form sub-themes and sub-themes were merged to generate major themes. Finally, the researchers proceeded with group-level analysis which was done by comparing the emerged subthemes and themes from the six groups in order to develop the major themes. This process was managed by NVivo software, version 11.

## Trustworthiness of the study

As soon as data was collected from a focus group, it was analysed and then the results were used to inform additional questions in the next focus group discussion to explore emerging themes. This concurrent data collection and analysis guaranteed that, emerging themes were explored in ensuing discussions. One instrument was used to conduct all group discussions which ensured consistencies. Discussions held in Twi (local Ghanaian language) were given to experts in that language to appraise so that transcripts were not misconstrued. This guaranteed correctness of the transcripts. The research team reviewed the themes to establish that all the data were included in the study. Field notes were used to verify and confirm the findings. Verbatim quotes were used to support the findings and this gave voices to the teenagers.

## Ethical clearance

All the participants gave consents before recruitment into the study. In addition, parents of participants below 18 years signed consent forms for their wards to sign assent forms. Ethical clearance was sought from the Institutional Review Committee of Ghana Health Service Ethics Review Committee (GHS-ERC: 07-11-2016). Approval was sought from participants before discussions were audio-taped. Participants were made aware that, participation in the study was voluntary and withdrawal at any time did not attract any form of penalty. The data presented had no form of identification that could be related to participants because codes were used to represent participants in the study.

## Results

### Participants’ background

Of the 30 participants, 8 maintained contact with their partners and 12 did not have any contact with their partners as they denied being responsible for the pregnancies. Ten of them were cohabiting with the men who impregnated them. The ages of the participants ranged between 13 and 19 years. Twenty had formal education (ranging from primary school to senior high levels) and 10 had no form of formal education. Twenty-one participants were unemployed and 9 were self-employed.

## Themes and sub-themes

Two major themes and eight sub themes emerged from the data after analysis. Please refer to Table 1.

**Table 1:** Themes and sub themes

Major themes	Sub-themes
Personal related barriers	Negative emotional feelings Irrational thinking Perceived health risks for the baby Perceived self-inefficacy
Social related barriers	Provider-client interaction Disapproval of exclusive breastfeeding by close relatives Unfriendly workplace policies Social myths

Source: Transcribed data

### Personal related barriers

Personal related barriers of exclusive breastfeeding as a major theme emerged after the following sub-themes were aggregated: *negative emotional feelings, irrational thinking, perceived health risks for the baby, and perceived self-inefficacy*.

#### Negative emotional feelings toward exclusive breastfeeding

Participants’ responded to the idea of exclusively breastfeeding their babies with *disbelief, anxiety, guilt, and defiance.* All these psycho-emotional responses were mainly based on the belief that breast milk does not contain water and enough nutrients for the survival of the babies.

*Disbelief* derived from the view that a baby is like any other human being. If an adult cannot survive without drinking water, how would a baby survive without water for six months?

“What is strange to me about exclusive breastfeeding is that, how can a human being who is alive be prevented from taking water? Me for instance I cannot stay from morning till evening without drinking water so how much more a baby?” **FG4B**^1^

“I cannot look at my child in the face and say that he should not drink water. Because even as an adult, when you are thirsty, imagine how you feel. How much more a small infant who does not know how to talk or do anything?” **FG3C**

Others could not imagine depriving their babies of water for six months after enduring so much during pregnancies.

“… The way I have suffered with this pregnancy, it would be very difficult to watch my child make “ta, ta, ta” sound in response to thirst and for her to be restless, I would have to give the water to the baby. Moreover, nine months journey of pregnancy is not easy and even as an adult, I know how it feels to be thirsty. I cannot watch my little baby to be lying there just like that without drinking water.” **FG4B**

*Anxiety* was linked to what would happen to their babies if they decide to exclusively breastfeed them for six months. They did not believe that breast milk contains all the required nutrients to maintain a baby for six months.

“As for that exclusive breastfeeding, it is no, no, no, I cannot give my child only breast milk for six months. I don’t know what would happen to my child when I choose to practice exclusive breastfeeding”. **FG2E**.

“It worries me when the nurses ask me to practice exclusive breastfeeding. Just the idea of not giving my child water or any other food than breast milk for six months pains my heart every time I hear of it.” **FG4A**.

*Guilty feeling* was mainly related to the participants’ beliefs about their responsibilities as mothers. They argued that as mothers, they will have the responsibility to take care of their children. One of this caring responsibilities is to ensure that the baby is comfortable and provided with the correct food. Exclusive breastfeeding was viewed as a failure to fulfil these responsibilities.

This feeling was best described in the context of a baby who is crying a lot with no apparent reason. For this group of participants, a baby who cries continuously while not sick, means that she/he needs additional water and food.

“If a baby is crying and that baby is not sick then surely, that baby is hungry or thirsty. As a mother, I would feel guilty if I do not give any food or water to the baby knowing that the baby is thirsty or hungry.” **FG1D**.

*Defiance* was expressed by the categorical refusal to exclusively breastfeed. For this group of participants, nothing would make them change their decision not to exclusively breastfeed their children.

“As for me, even if my head is hit on the floor or even if I am sent to the shrine, I would give my baby water to drink right from birth. Just that I would have to keep the water clean.” **FG5B**

“… when the nurses talk to me about exclusive breastfeeding, I would not say a word because the nurse is not in my head and would also not follow me home. If I deliver my baby and notices that the reaction suggest thirst, then I would give water to him/her even if the baby is one day old.” **FG4C**

#### Irrational thinking towards exclusive breastfeeding

This irrational thinking was related to both the benefits and the outcome values of exclusive breastfeeding. Participants were aware of the benefits of exclusive breastfeeding but remained sceptical about the expected outcomes. They questioned the benefits of exclusive breastfeeding as illustrated with the extracts below:

“Which child would say that he or she would not love you? Every child would love the mother whether the child is breastfed exclusively or not. Even if the child is not given any food by the mother, that child would love the mother. As for that one, the nurses say it just for saying sake.” **FG6A**

Some described breastfeeding education as ‘nonsense’. They listened to those ‘nonsenses’ only when they were at the hospital.

“I h﻿ave been telling myself that -you nurses can continue speaking about exclusive breastfeeding because it is your work. They are looking after my health, so I cannot disagree with them overtly or tell them to stop the nonsense they are saying about feeding the baby breast milk alone for six months. When I get home with my baby, I know exactly the kind of wholesome water and food I will give to my baby from day one.”**FG4B**.

Others hilariously said that they always felt like physically slapping nurses when they talk about exclusive breastfeeding.

“When the nurses are talking about exclusive breastfeeding, I sometimes wish I could slap their mouths for saying that. If I had the chance to slap her lips, I would have really done it. The bottom line is that, I would not agree to practice exclusive breastfeeding (LAUGHING).”**FG4A**.

Intellectualisation was another irrational thinking used by the participants to justify the refusal to exclusively breastfeed. Participants argued that exclusive breastfeeding should only be practiced by mothers with poor hygiene and those who lack knowledge on how to prepare and maintain the baby’s’ feeding bottle.

“The reason why they tell us to exclusively breastfeed our babies is because of mothers who do not know how to keep the feeding bottles of the babies neat. But if a mother knows how to prepare a baby’s feeding bottle and to keep it clean, she should not consider exclusive breastfeeding.” **FG3C**.

“The most important thing in this issue is to give the child wholesome water and to keep the baby’s feeding bottle clean and free from dirt. I think that, once that is done, then the mother can give water to the baby right from birth.” **FG4D**.

Few would not understand why they are expected to feed their babies only with breast milk for the first six months while they cannot even afford nutritious foods that can stimulate the production of breast milk.

“As my sister said, the whole thing is about affordability. After delivery, if the man runs away and abandons the mother and baby, it would be difficult for the mother to afford the right food which would help in the production of breast milk for the baby. Hmmm, it is a problem oh….” **FG3C**

“Food is too expensive here. To prepare small soup, I spend a lot. Meanwhile, I am a student and the father of the child whom I am carrying has abandoned me. So how can I afford the food that can enhance the production of breast milk to allow me to breastfeed?” **FG4D**

#### Perceived health risks for the baby

Participants believed that exclusive breastfeeding *is life threatening, increases the risk for diseases, and slows the development and growth* of the baby.

*Life threatening* was derived from the belief that breast milk does not contain water and enough nutrient, therefore depriving the baby of additional water and food in the first six months increases the chance of that baby to die.

“I tell myself that breast milk is good but I also sometimes think to myself that if a baby is not given any water for six months, then *that baby can die*.” **FG1D**.

“A baby cannot live on only breast milk for six months. If the baby does not take in foods other than breast milk, the baby cannot live and would die. So although a mother would give breast milk, she must add food and water as well.” **FG2C**.

Some believed that exclusive breastfeeding increases the risk of a baby to contract diseases such as HIV and cancers.

“Sometimes, if you give only breast milk to the baby, it can make the baby fall sick” **FG2B**.

“Sometimes some of the mothers have certain diseases like cancer in their breasts and HIV AIDS. So if such a mother is asked to breastfeed exclusively for six months, she can transmit such diseases to the baby.” **FG3D**.

They also believed that exclusive breastfeeding exposes a child to the risk of developing eating disturbances. They believed that if you don’t introduce additional food early enough, the child will grow-up hating food or developing eating problems.

“Exclusive breastfeeding will make my child hate food or develop eating problems when he grows-up. So, I would like my child to learn how to eat from birth so that I can prevent her from developing problems with eating when he grows-up. That is why it would be difficult for me to practice exclusive breastfeeding.” **FG6B**.

#### Perceived self-inefficacy

Perceived self-inefficacy as a barrier to exclusive breastfeeding refer to things or circumstances that are viewed as negatively affecting the personal capabilities of the participants to exclusively breastfeed their babies. They attributed their incapability’s to exclusive breastfeeding to the *negative effect on self-body image, vulnerability to diseases, and reduce personal income and development*.

*Negative effect of exclusive breastfeeding on self-body image;* participants believed that exclusive breastfeeding or breastfeeding in general makes mothers unattractive because of changes in the shape of their breast and weight. Some believed that their breast would ‘sag’ if they breastfeed their babies.

“If a woman allows a baby to suck too much breast milk, the breast would sag too much and you will start looking like a boy. That is the reason why some of us will not give breast milk to our babies.” **FG6C**.

“The breast would sag and when the breast sags, you lose your beauty and no man would be attracted to you.” **FG6A**.

Others believed that a mother who practices exclusive breastfeeding would lose too much weight, which has negative impact on their self-body image.

“Please as for me, the little I know which I can say is that, breastfeeding would also make you lose too much weight to the extent that your body shape will completely change. …Boys will no longer be interested in you. Some may think that you have AIDS.” **FG2E**.

*Vulnerability to diseases;* participants believed that breastfeeding makes mothers vulnerable to certain diseases such as *high blood pressure.*

“You know that mothers who feed their babies with breast milk only for six months are at high risk of developing high blood pressure. Because of the disturbance in their sleep patterns as they have to wake up even at night to breastfeed their babies.” **FG1D**.

Others believed that exclusive breastfeeding can expose mothers’ breast to spiritual ‘bad eyes’ that can afflict them with all types of diseases in the breast.

“Some people have “bad eyes” when you expose your breast, they can put some diseases into the breast. Sometimes, when the breast is exposed, someone can look at it and make the breast swell. In that case, that person has given you a disease in the breast. That makes practicing exclusive breastfeeding difficult.” **FG6D**.

*Reduce personal income and development;* participants believed that the duration required to *e*xclusively breastfeed their babies would negatively affect their income and compromise their personal development plans.

“The six months exclusive breastfeeding is too much. It would not allow the mother to work to earn money in order to take care of the child and herself.” **FG1B**.

Personal development plans were quoted by participants who were looking forward to returning to school after delivery.

“For some of us who want to go back to school, exclusive breastfeeding would inconvenience us. I would have to stay at home for at least six months, which means a year of not schooling. You may no longer have interest and motivation to go back to school after staying home for a year. So, what type of future would I have without education?” **FG2A**

### Social related barriers

Social related barriers of exclusive breastfeeding emerged from the participants’ views regarding the influence of the *provider-client interaction, disapproval of exclusive breastfeeding by close relatives, unfriendly workplace policies and social myths.*

A person’s perception of the social environment as unsupportive to the behaviour and the belief of the disapproval of the behaviour by significant others may prevent them from engaging in that behaviour [36].

#### Provider-client interaction

The nature of the interaction between the provider (nurses and midwives working at antenatal care) and the client was perceived as a barrier to exclusive breastfeeding. This interaction was described by the participants as unfriendly and hostile. This behaviour prevented them from seeking information that would have allowed them to make informed decision regarding exclusive breastfeeding. This negative behaviour of the nurses and midwives toward the participants can be attributed to the social prejudice attached to teenage pregnancy in the country.

“Eii, as for me, I cannot tell a nurse what is truly on my heart oh. I am even afraid to ask any questions to a nurse. They would shout at you before you even finish talking. They call you all these names for being pregnant. It makes it so difficult for one to decide on exclusive breastfeeding or not.” **FG6C**.

“How would you expect me to even consider feeding my baby with breast milk when I am so scared to ask my midwife questions about it? She is too harsh oh. If you miss an appointment, you would be in trouble the next time you show up. She would ask you to sit and wait till the last person leaves before she would take care of you. But she does not treat other women the same way.” **FG6B**.

#### Disapproval of exclusive breastfeeding by close relatives

Participants would not consider exclusive breastfeeding if their family members do not support the idea of exclusively breastfeeding a child irrespective of the nurses’ advice.

“You know what, my mother does not support feeding a baby with breast milk only for six months. So, I will add water and give some food as she did with us.” **FG2B**.

“All my sisters do not support this idea of exclusive breastfeeding. I support what my nuclear family say about exclusive breastfeeding. Some food and water should be added to the baby’s meal right from the beginning.” **FG1D**.

The influence of the mothers was so strong that the choice of the participants did not matter.

“Me for instance when I give birth, no matter what I do whether I like it or not, my mother would give water to the baby. Because she would say that water for babies is good. She has already started telling me that when I deliver, water would be the first thing which she would give to the baby. She says that, water gives the babies strength so water must be given to the baby immediately after delivery.” **FG4D**.

#### Unfriendly institutional policies

For working and schooling participants, existing policies were not promoting exclusive breastfeeding. The duration of maternity leave and inflexible workplace policies were viewed by working participants as negatively influencing their attitudes towards exclusive breastfeeding.

“A working mother cannot exclusively breastfeed her baby. The work policies do not allow a mother to stay at home for a long time after delivery and there are no provisions to allow mothers to take time off to breastfeed their babies during working hours. So, there is no way we can consider exclusive breastfeeding during the first six months.” **FG1D**

Others attributed negative exclusive breastfeeding attitudes to the schedule and types of jobs they were doing.

“I report to work at 6am. If my boss would agree that I report to work at 8am instead of 6 am, I would be able to practice exclusive breastfeeding. If he would also allow me to bring my baby along to the workplace, it would also make it easy for me to practice exclusive breastfeeding. If he could also reduce the workload for me and increase my salary, it would be easier for me to practice exclusive breastfeeding.” **FG1A**.

“Personally, I am a hairdresser and I am asked to breastfeed exclusively for six months. When I’m at work and a customer approaches me that she wants to style her hair, do you think that I should tell the customer to wait for me to go give my baby breast milk? This means that the customer will go to another saloon and before I know it, my boss would dismiss me.” **FG3B**.

School policies were raised specifically by the participants who planned to go back to school after delivery. As with the workplace policies, the school policies do not make provision for the learners to take time off to go breastfeed their babies during schooling.

“I have to go back to school after delivery and there is no way I can exclusively breastfeed my baby for even a day. I cannot take my baby to school because there is no place where I can leave her when attending classes. In addition, the school will not even allow me to go breastfeed my baby during classes.” **FG2A**

#### Social myths

Social myths about exclusive breastfeeding as perceived barriers of exclusive breastfeeding refer to social values or beliefs attached to exclusive breastfeeding that were viewed by the participants’ as negatively influencing their desire to exclusively breastfeed their babies. These beliefs and myths were based on the *social value attributed to exclusive breastfeeding and social misconceptions about exclusive breastfeeding.*

From the behavioural perspective, an individual’s perception of the social values or beliefs surrounding a behaviour may positively or negatively influence their intentions to perform that particular behaviour [36].

*Social value attributed to exclusive breastfeeding;* exclusive breastfeeding was equated to low social standing in the community. Mothers who exclusively breastfeed their infants for six months were viewed as poor and regarded as of low social class.

“The people in the community would say that I am poor if I decide to practice exclusive breastfeeding. They would assume that I am extremely poor and that I cannot afford the baby food from the shop. So to avoid their criticisms, I would have to add food and water to the breast milk when my baby is born.” **FG6B**.

“In the village for instance, they would be saying that I am practicing exclusive breastfeeding because I am poor and cannot afford the baby food. To avoid that, I will not practice any form of exclusive breastfeeding.” **FG6C**.

*Social misconception about* exclusive breastfeeding; this barrier refers to the perceptions of the community members, specifically, older women. They believed that exclusive breastfeeding makes a new born baby cry at night or to continuously cry during the day.

“Most people do not expect a baby to cry at night or cry a lot during the day. If this happens, they would talk negatively about you and the older women would often ask you: ‘aren’t you giving your baby water? You must give him water and food’ and they will really talk. So I won’t practice exclusive breastfeeding.” **FG4B**.

## Discussion

Two major themes and eight sub themes emerged from the data after analysis. Personal related barriers (negative emotional feelings, irrational thinking, perceived health risks for the baby, and perceived self-inefficacy) and social related barriers (provider-client interaction, disapproval of exclusive breastfeeding by close relatives, unfriendly workplace policies and social myths) were the perceived factors that discouraged exclusive breastfeeding among teenage mothers.

Most participants’ expressed negative emotional feelings and irrational thoughts about exclusive breastfeeding practice for six months. This could be linked to the fact that most teenage pregnancies are unplanned and the practice of exclusive breastfeeding has been positively associated with planned pregnancies in Lebanon[37], Philippines [38], and Kenya [39]. Although fear has been reported among breastfeeding mothers with HIV in Ghana [40], it has mostly not been reported among teenage mothers. It may be possible that, all the above negative feelings about exclusive breastfeeding expressed by the teenagers in this study may be as a result of negative stories heard in their communities about the effect of exclusive breastfeeding on the baby. A lot of work needs to be done in the communities by nurses and midwives, to help change perceptions about the effect of exclusive breastfeeding on the mother, baby and the community as a whole. Perhaps nurses and midwives can only change perceptions if they are given provider training on cultural sensitivity so that, they will avoid criticisms and negativity which can encourage the teens to ask questions during antenatal clinic. If perceptions are not changed at the community level, all efforts at improving exclusive breastfeeding among teenage mothers may have little impact.

With regards to perceived health risks of exclusive breastfeeding to their babies, a lot of misconceptions were expressed. Some participants believed that exclusive breastfeeding could cause health risks including death among infants. This current finding is similar to that of several studies whereby misconceptions and beliefs about breastfeeding affect breastfeeding practices [15, 41–43]. Perhaps these misconceptions are indications that, most teenage girls are misinformed and uneducated about issues concerning breastfeeding practices. Even among female educated college women, inadequate information and misconceptions about breastfeeding were the main reasons behind negative attitudes towards breastfeeding [44]. This buttresses the need for further education of all the stakeholders in breastfeeding because some of these misconceptions are fuelled by close relatives like grandparents of the infants [41]. Meanwhile, women who receive adequate information about breastfeeding have been documented to be more likely to breastfeed compared to their counterparts who are ignorant about the subject [44–49]. Meanwhile, it has been reported that, women who are comfortable breastfeeding in public have positive attitudes towards breastfeeding [44, 48, 50, 51]. Therefore, breastfeeding friendly attitudes should be encouraged in public for teenage mothers to feel at ease when breastfeeding in public.

Perceived self-inefficacy was expressed by participants as circumstances that are viewed as negatively affecting the personal capabilities of the participants to exclusively breastfeed their babies. This finding is consistent with other studies where the mothers were not confident enough in the ability of breast milk to provide sufficient nutrients without creating other problems for them and their infants [39, 41–43]. In addition some participants were of the view that, exclusive breastfeeding would thwart their finances and personal development since it consumes time, prevents them from engaging in economic activities as well as prevents them from returning to school. This is similar to a study where the mothers were of the view that, in the immediate short term, breastfeeding reduced their personal income since they could not put in as many hours at work as desired [52]. Meanwhile, the long term global cost for not breastfeeding has been estimated to be about $302 billion annually [53]. Perhaps the participants in this study were only interested in the short term economic loses without paying attention to the long term economic benefits of breastfeeding. A study at Kenya has also reported similar findings where young mothers could not practice exclusive breastfeeding due to their desire to return to school [15]. Measures should be put in place by healthcare professionals and teachers to encourage breastfeeding. Teachers should be trained on how to handle teenage mothers in their classrooms in order to promote breastfeeding. Participants also expressed concern about the possibility of their breasts sagging for them to become unattractive if they practiced exclusive breastfeeding. Sagging breast as a deterrent for optimal breastfeeding has been reported widely in literature to an extent that, even lactating nurses and midwives also have concerns about their breast sagging after breastfeeding [54–57]. With this information, antenatal education should persistently be tailored to address body image issues.

Health provider-client interaction and disapproval of exclusive breastfeeding by close relatives were causing conflicting views in the minds of some participants. Health professionals were reported to be harsh and unfriendly which prevented participants from asking nagging questions about breastfeeding and that gap was filled by close relatives who were providing wrong information about breastfeeding. Therefore, there were lots of scepticisms expressed about what health professionals said about exclusive breastfeeding by participants. Most of them believed what their mothers had said about exclusive breastfeeding more than what the nurses had told them. This finding is largely contrary to a study which has reported that most women (≥ 18 years) took the advice of their health professionals on breastfeeding issues [58]. Conceivably, the mode of delivery of breastfeeding messages at the hospitals and the brief contact participants had with their midwives during each antenatal visit eluded them of personal interactions. This finding is specially worth paying attention to since it has emphasized the need for midwives attending to teenage pregnant girls to work with both the pregnant teenagers and their mothers to change negative perceptions about exclusive breastfeeding. The nurses and midwives should also be trained on how to relate with pregnant teenagers in order to avoid prejudices.

The role of institutional policies in influencing the decision of mothers to exclusively breastfeed their children is documented in previous studies. The existence of workplace policies perceived as baby-friendly by mothers were attributed to positive attitudes towards exclusive breastfeeding [59, 60]. Mothers who have been found to work at places with friendly work policies for breastfeeding mothers have been able to breastfeed successfully for longer duration [61]. This may be due to the fact that exclusive breastfeeding is time consuming and demanding. Therefore, policies about work should be such that, breastfeeding mothers are given shorter working hours, longer breaks and maternity leaves which would go a long way to promote exclusive breastfeeding.

Participants reported social myths about breastfeeding where they believed that, exclusive breastfeeding in their communities was a sign of poverty and breast milk was not enough to satisfy infants which leads them to cry often. With regards to the misconception that, breast milk alone is not enough for infants, it has been reported by other studies [15, 62]. Perhaps, the expressed myths is as a result of ignorance on the part of the participants. These myths and misconceptions about breastfeeding may be debunked through intensive media breastfeeding campaigns.

The study was limited to pregnant teenagers within the Greater Accra Region of Ghana. Other studies can focus on pregnant teenagers in other parts of Ghana since different cultural dynamics may bring out different perspectives.

## Conclusion

Personal related barriers (negative emotional feelings, irrational thinking, perceived health risks for the baby, and perceived self-inefficacy) and social related barriers (provider-client interaction, disapproval of exclusive breastfeeding by close relatives, unfriendly workplace policies and social myths) were the perceived factors that discouraged exclusive breastfeeding among teenage mothers. Such factors could be confronted by health professionals, family members (especially mothers of pregnant teenagers), policy makers and the society as a whole in order to promote exclusive breastfeeding among teenage mothers. The Ghana Health Service together with other stakeholders should design tailor made provider training on cultural sensitivity for all health professionals that cater for teenagers. In addition, a collective effort from all stakeholders including the media, religious leaders, politicians and non-governmental organizations are needed to be able to debunk such misconceptions that were expressed by participants in this study.

## Data Availability

The datasets analysed during the current study is available through the corresponding author on reasonable request.

## Footnotes

1 Focus groups were labeled from 1 to 6 and participants within each focus group were labeled A to E. So for example; FG4B means: second participant within the fourth focus group.

## Declarations

**Ethics for Approval and consent to participate**.

Ethical clearance was sought from the Institutional Review committee of Ghana Health Service Ethics Review Committee (GHS-ERC: 07-11-2016). Informed consent was obtained from all participants who were 18 years and above. In addition, informed consent was obtained from parents of participants below 18 years while informed assent was also obtained from participants below 18 years. Since this study involved humans, it was conducted in accordance with international ethics guidelines or Declaration of Helsinki.

## List of abbreviations

AIDS: Acquired Immune Deficiency Syndrome
WHO: World Health Organization
